# EStiMapp: A practical tool for mapping clinical symptoms evoked by electrical stimulation in intracranial EEG for epilepsy surgery

**DOI:** 10.1016/j.cnp.2026.06.006

**Published:** 2026-06-12

**Authors:** Irene B. Heijink, Susanne B. Jelsma, Sandra M.A. van der Salm, Nicole E.C. van Klink, Cyrille H. Ferrier, Sem Hoogteijling, Charlotte J.J. van Asch, Dorien van Blooijs, Maeike Zijlmans, on behalf of the RESPect database group

**Affiliations:** a Department of Neurology and Neurosurgery, University Medical Center Utrecht Brain Center, University Medical Center Utrecht, Full Member of European Reference Network EpiCARE, P.O. box 85500, 3508, GA, Utrecht, the Netherlands; b Stichting Epilepsie Instellingen Nederland (SEIN), P.O. box 540, 2130, AM, Hoofddorp, the Netherlands

**Keywords:** Focal epilepsy, Intracranial EEG, Electrical stimulation, Clinical symptoms, Evoked symptoms, Graphical user interface, Co-design

## Abstract

**Objective:**

Electrical stimulation of intracranial electrodes can map the patient's specific functional regions and seizure related symptoms. In our clinical practice, evoked functional symptoms and electrographic responses are manually annotated and visualized in a schematic electrode overview: an error-prone and time-consuming process. We here present an open-source graphical user interface (GUI) that standardizes the workflow and automatizes the visualization of intracranial EEG (iEEG) electrical stimulation results.

**Methods:**

We defined categories of evoked clinical symptoms based on a literature study and consensus session. A co-design team participated in brainstorm sessions to set the requirements of the GUI. We built the GUI and tested its usability with qualitative and quantitative assessments (System Usability Scales (SUS) questionnaires).

**Results:**

The workflow included standardized annotation of categories of evoked clinical symptoms in the iEEG software. The GUI visualized these evoked clinical symptoms in a 2D schematic overview, a 3D visualization of the brain with locations of evoked symptoms, and showed additional annotations per stimulated electrodes in a table. A mean SUS score of 82.3 (SD 9.2) was reached, which is considered excellent.

**Conclusions:**

We present an in-house developed workflow and graphical user interface (GUI) to categorize and directly visualize evoked clinical symptoms resulting from electrical stimulation on intracranial electrodes.

**Significance:**

Our open-source GUI facilitates standardization of the intracranial stimulation workflow and directly enables straightforward and uniform interpretation of evoked clinical symptoms.

## Highlights

• We present an open-source tool for mapping stimulation evoked symptoms in iEEG.
• A co-design team collaborated to standardize the workflow in a time-saving manner.
• Seven out of eight users deemed the tool more useful than standard clinical work-up.

## Graphical abstract

**Figure:**
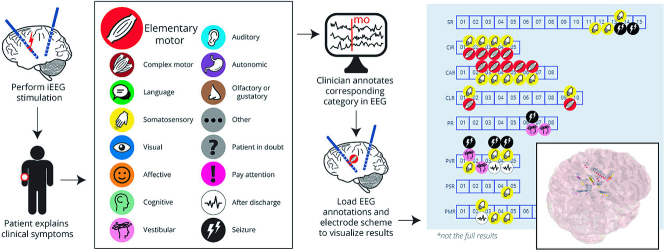

## 1 Introduction

Epilepsy surgery can cure people from an otherwise lifelong disease. As part of their presurgical evaluation, people with refractory focal epilepsy might undergo intracranial electroencephalography (iEEG), either with stereo EEG electrodes (sEEG) or with subdural grid electrodes (SDE). The aim of iEEG is to record seizures to localize the seizure onset zone (SOZ) and to distinguish eloquent brain areas (Guery and Rheims, 2021). During iEEG monitoring periods, electrical stimulation of the intracranial electrodes can unveil information about the patient's specific location of function, seizure related symptoms, and connectivity in the brain. This information can aid in decision making for determining the resection area during presurgical evaluation. In our current clinical standard practice, patients describe and clinicians observe the clinical symptoms evoked during stimulation which are then annotated as free text within the EEG software and on paper. Afterwards, the clinical symptoms and electroencephalographic responses are manually visualized in a schematic electrode overview in Excel or Word and then put in the clinical report: an error prone and time-consuming process. The current unstructured input and user-dependent visualization creates diversity in electrode schemes making it difficult to cooperate among clinician and align large sets of data for research or clinical lessons.

Recent advancements in technology can aid in mapping out, monitoring and automating workflows and thereby saving time of clinicians. Examples of such advancements are medical dashboards, graphical user interfaces (GUIs) and apps (Almasi et al., 2023; Kopanitsa et al., 2013). The epilepsy surgery trajectory is time-depending and the specific workflows can benefit much from these new technologies. In this article, we focus on mapping clinical symptoms and electroencephalographical responses evoked during the iEEG electrical stimulation workflow.

For a structured input to automate the iEEG electrical stimulation workflow, guidelines on classification of evoked symptoms are needed, but currently lacking (Fisher et al., 2017). Existing work focused primarily on spontaneous aura and epileptic seizure types (Asadi-Pooya et al., 2016; Foldvary-Schaefer and Unnwongse, 2011) or only language and motor mapping (Cuisenier et al., 2020; Trébuchon and Chauvel, 2016). Our goal is to give an overview of all categories of evoked clinical symptoms that is easy to interpretate. Previous work on the visualization of iEEG data lack the ability to process electrical stimulation mapping (Del Vecchio et al., 2024; Groppe et al., 2017; Lin et al., 2019; Lucas et al., 2024; Trotta et al., 2018). For example, IntrAnat, YAEL and RAVE offer the option to visualize categorical iEEG data, but still require manual definition of the categories of evoked symptoms (Deman et al., 2018; Magnotti et al., 2020; Wang et al., 2023). gTEC cortiQPRO offers an electrical stimulation mapping tool, but requires the purchase of their software and hardware system (cortiQ Rapid Cortical Mapping | g.tec medical engineering, 2026).

We present an open source clinical GUI to categorize and visualize evoked clinical symptoms and electroencephalographical responses. This GUI facilitates rapid visualization of categorical evoked clinical symptoms, and enables straightforward review of annotated comments. Prior to the design of the GUI, we performed a literature study to define categories of evoked clinical symptoms. With clinical stakeholders, we ensured that the GUI was aligned with their needs and preferences to optimize the clinical usability of the tool. This article describes the co-design process and usability testing, the selection of clinical symptom categories, and the presentation of the open-source GUI. Our aim is to standardize, visualize and accelerate the iEEG electrical stimulation workflow.

## 2 Methods

### 2.1 Categories of evoked clinical symptoms

**Fig. 1:**
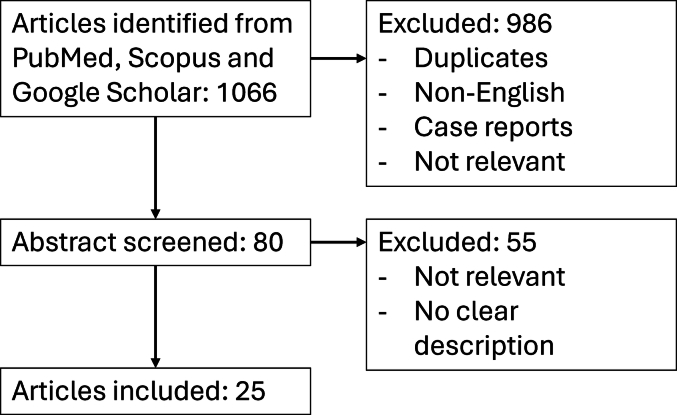
Flowchart of the literature search.

A comprehensive narrative review was conducted to synthesize an overview of categories of evoked clinical symptoms. The electronic databases PubMed, Scopus, and Google Scholar were searched using the following terms as keywords: “aura”, “function”, “mapping”, “stimulation”, “intracranial EEG”, “epilepsy” and multiple variants of these terms. Study inclusion criteria were as follows: written in English, full text available, studying humans, clear description of categorized clinical symptoms listed, and covering a range of clinical symptoms including auras. The search was performed without restricting publication dates at the 13th of June 2025. A full-text review was performed by IH when the screened abstract was considered relevant. We removed duplicates and searched reference lists of included articles to ensure comprehensive literature retrieval (see Fig. 1). We included classifications based on electrical stimulation, spontaneous seizures, or both. We selected categories that were common practice, aligned with the ILAE guidelines for seizure classification (Beniczky et al., 2025), and prevalent during electrical stimulation in iEEG. The selection resulted in a list of categories, a description of the clinical symptoms per category, an abbreviation per category used for annotation, and an icon for visualization in the GUI.

### 2.2 Co-designing the graphical user interface

The Coproduction Design and Implementation Flow Model (CDIFM) was applied to co-design the GUI. The CDIFM is a user-centered framework that is regularly used in co-designing medical dashboards (Morken et al., 2024; Perry et al., 2022). The four steps of the co-design process were: 1) define the problem of the end-users, 2) understand the context of use and lived experience, 3) build a design consensus, 4) establish design specifications and do pilot-testing.

We established a co-design team with three clinical neurophysiologists and three technical physicians who perform intracranial electrical stimulation in the University Medical Center (UMC) Utrecht. Technical physicians are healthcare professionals with knowledge and skills in the fields of both medicine and technology (The NVvTG – NVvTG.nl, 2026). These end-users were involved to ensure practical usefulness of the GUI. We organized four brainstorm sessions with the co-design team between September 2024 and August 2025 to iteratively develop the GUI passing the four co-design stages (see Appendix A). Data sources included recordings and notes from co-design meetings and user interviews, and questionnaires.

Usability testing of the GUI was performed using qualitative assessment, and quantitative assessment with a questionnaire including the System Usability Scale (SUS) (Brooke, 1996). The questionnaire can be found in Appendix B. The SUS is a scale from 0 to 100 by the co-design team and external end-users respectively. A score above 80 is generally considered ‘excellent’, a score above 68 is considered ‘good’. Both the co-design team and five independent end-users took part in testing the final version of the GUI and filled in the questionnaire. The independent end-users were four clinical neurophysiologists and one physician assistant from University Medical Center Utrecht, Stichting Epilepsie Instellingen Nederland, and The Swiss Epilepsy Center at Klinik Lengg, Zürich.

### 2.3 Workflow using the graphical user interface

**Fig. 2:**
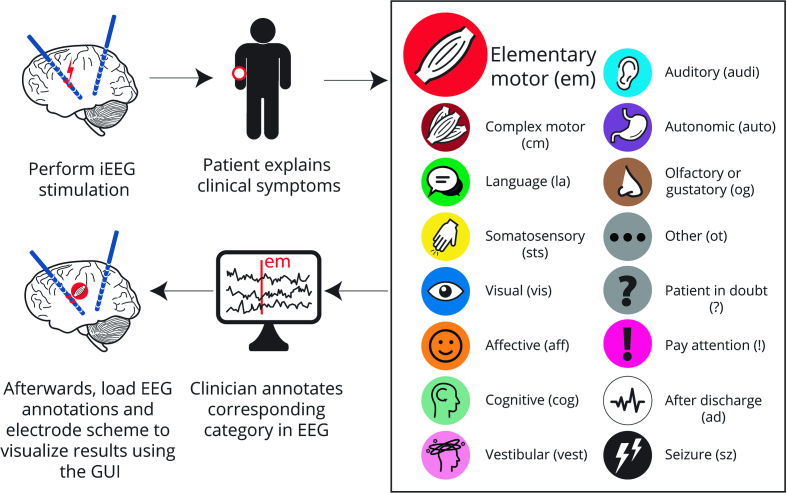
Workflow using the GUI. The abbreviations of the categories of evoked clinical symptoms are used to annotate the category in the iEEG software.

The workflow comprehended several steps (see Fig. 2). First, the clinician performed electrical stimulation functional mapping and annotated evoked clinical symptoms and the corresponding abbreviated categories of symptoms in the iEEG software. The annotations were exported from the iEEG software. Then, the input data is uploaded into the GUI. The input data consisted of a tabular file (xlsx) with the patient specific electrode scheme indicating all implanted electrodes including channel numbers, and a tabular file (CSV) with the exported iEEG annotations during electrical stimulation. Optional 3D visualization required a PLY file of the cortex and a tabular file (xlsx) containing the electrode names, number of contacts, and entry and target coordinates of the electrodes. After uploading the files, the GUI displayed the results automatically on the patient specific electrode configuration in 2D and 3D using category specific icons. In addition, a table containing all annotations per stimulation pair was shown. All stimulation parameters as recommended in the technical standards by Arya et. Al (2025) can be shown in the column ‘stimulation type’. These stimulation parameters include polarity, phases, pulse frequency, pulse width, train duration, and current intensity (Arya et al., 2025). The end-user can edit the category and notes, and download the table as CSV. The visualization was exported as PNG to be inserted in the clinical report. A step-by-step guideline can be found in the supplementary material S1.

The GUI was evaluated by the UMC Utrecht's internal advisory committee for classification of medical devices, which classified the device as presented as non–medical device software, thus not falling within the scope of the EU MDR. The GUI was developed as a web application based on Python 3.13 using Dash Mantine. The GUI can be installed and run locally using the code available on Github, or online using the web application hosted on Render. Please be aware that for the latter, data is uploaded to the virtual environment of Render. Data is not stored in the application. Data can be processed anonymously. The required functionality of the GUI is shown in Table 1.

**Table 1:** REQUIREMENTS OF THE GUI.

*General*	1	Load the intracranial electrode scheme
	2	Load the annotations
	3	Link the annotations of evoked categories of clinical symptoms to the stimulated electrodes
	4	Process data locally and/or anonymously
*2D visualization*	5	Calculate the coordinates of the categorized evoked clinical symptoms on the 2D electrode scheme
	6	Load the category specific icons of the evoked clinical symptoms
	7	Project the icons on the 2D electrode scheme
	8	Display the 2D figure and legend
	9	Option to save the 2D figure
*3D visualization*	10	Load the entry and target electrode coordinates
	11	Load the 3D PLY object
	12	Calculate the coordinates of the electrode contacts
	13	Project the electrode contacts on the 3D rendering of the brain
	14	Calculate the coordinates of the categorized evoked clinical symptoms on the 3D rendering
	15	Project the category specific colored markers on the 3D rendering of the brain
	16	Display the 3D figure and legend
	17	Option to save the 3D figure
*Table*	18	Make a table with stimulated electrodes, categories of evoked clinical symptoms, free text annotations, and stimulation type
	19	Display the table
	20	Option to save the table

### 2.4 Data and code availability statement

The source code and web application are publicly available on GitHub (https://github.com/UMCU-EpiLAB/umcuEpi_estimapp.git) and (https://estimapp.onrender.com). The data of one example patient with stereo EEG is available on DataverseNL (https://doi.org/10.34894/KMT3VI). The example patient provided informed consent for data use via the Registry for Epilepsy Surgery Patients (RESPect database) of the University Medical Center Utrecht (UMCU) and Stichting Epilepsie Instellingen Nederland (SEIN). The Medical Ethical Committee of the UMC Utrecht approved collecting pseudo-anonymized data in the RESPect database.

## 3 Results

### 3.1 Categories of evoked clinical symptoms

Twenty-five articles were included in the narrative synthesis of our literature search, see Fig. 1. The clinical symptoms categories per article are shown in Table 2. Ten articles based their categories on iEEG stimulation data, twelve articles on spontaneous seizures, and three articles used both iEEG stimulation data and spontaneous seizures. Four of the articles based on iEEG stimulation data focused on language mapping (Cuisenier et al., 2020; Jahangiri et al., 2021; Reecher et al., 2024; Wen et al., 2017). In two of seven articles, the category motor was separated into simple and complex (Beniczky et al., 2025; Foldvary-Schaefer and Unnwongse, 2011). In two of seven articles, the category language was divided into comprehension and production (Cuisenier et al., 2020; Wen et al., 2017). In six of seven articles, the categories olfactory or gustatory were separated into two categories (Ashalatha et al., 2018; Beniczky et al., 2025; Foldvary-Schaefer and Unnwongse, 2011; Nakken et al., 2009; Perven and So, 2015; Russo et al., 2019). These subcategories are indicated with an asterisk in Table 2. Categories that were excluded in this study are: abdominal/epigastric (Asadi-Pooya, 2021; Asadi-Pooya et al., 2016, Asadi-Pooya et al., 2016; Ashalatha et al., 2018; Foldvary-Schaefer and Unnwongse, 2011; Halgren et al., 1978; Russo et al., 2019; Yin et al., 2021; You et al., 2023), automatisms (Nakken et al., 2009; Salanova et al., 1992), sexual (Ashalatha et al., 2018; Russo et al., 2019), cephalic (Asadi-Pooya, 2021; Ashalatha et al., 2018; Foldvary-Schaefer and Unnwongse, 2011; Palmini and Gloor, 1992; Yin et al., 2021), and multiple (Asadi-Pooya et al., 2016; Ashalatha et al., 2018; Foldvary-Schaefer and Unnwongse, 2011; Russo et al., 2019; You et al., 2023). We grouped ‘abdominal/epigastric’ together with ‘autonomic’, ‘automatisms’ together with ‘complex motor’, and ‘sexual’ together with ‘affective’. Instead of one category ‘multiple’, all evoked categories could be indicated at one stimulation pair.

**Table 2:** LITERATURE OVERVIEW OF CATEGORIES OF CLINICAL SYMPTOMS.

												
	Elementary motor	Complex motor	Language	Somatosensory	Visual	Affective	Cognitive	Vestibular	Auditory	Autonomic	Olfactory or gustatory	Other
Based on electrical stimulation:												
Aungaroon et al., 2023				x								
Cuisenier et al., 2020			x*									
Halgren et al., 1978					x	x	x					
Jahangiri et al., 2021	x		x	x								
Jaroszynski et al., 2022									x			
Reecher et al., 2024			x									
Salanova 1995b	x		x	x	x			x				
Zea Vera et al., 2017	x		x									
Wen et al., 2017			x*		x				x			
Yu et al., 2018	x			x	x			x	x			
Based on spontaneous seizures:												
Asadi-Pooya et al., 2016						x	x					x
Asadi-Pooya et al., 2016					x	x	x					x
Asadi-Pooya, 2021				x	x	x	x	x	x		x	x
Beniczky et al., 2025	x	x	x	x	x	x	x	x	x	x	x*	x
Nakken et al., 2009	x			x	x		x	x	x	x	x*	
Palmini and Gloor, 1992				x	x		x	x	x	x		
Perven and So, 2015				x	x			x	x	x	x*	
Radhakrishnan 2018				x		x	x	x	x		x*	x
Russo et al., 2019					x	x	x	x		x	x*	
Salanova et al., 1992					x							
Yin et al., 2021				x	x	x	x	x	x	x		x
You et al., 2023				x		x				x		
Based on electrical stimulation and spontaneous seizures:												
Duong et al., 2025				x								
Foldvary-Schaefer and Unnwongse, 2011	x	x		x	x	x	x	x	x	x	x*	
Yan et al., 2024					x			x		x		

The asterisk indicates that the authors separated this category in subcategories: language in comprehension and production, and olfactory or gustatory in olfactory and gustatory.

Based on this literature study, we proposed categories of clinical symptoms to the co-design team and reached consensus defining the following categories: motor, language, vestibular, autonomic, affective, cognitive, visual, auditory, somatosensory, olfactory or gustatory, and other. See Table 2. The category other contains all evoked clinical symptoms that could not be classified, for example indescribable auras. Besides the classification of clinical symptoms, we added some extra note types: patient in doubt, pay attention, after discharge, and seizure. ‘Patient in doubt’ was used when the patient description of a symptom is unclear. ‘Pay attention’ was used when the evoked symptom is unexpected at this anatomical location.

### 3.2 Co-designing the graphical user interface

The primary outcome of the co-design process was a fully functional GUI that could be implemented in clinical practice, as shown in section 3.3. The secondary outcome was the quantitative and qualitative data regarding usability testing. Output of the co-design meetings and user interviews was processed to develop the GUI further. The co-design process was concluded in October 2025.

The GUI's usability was tested during stimulation of six sEEG patients. On average, four categories were found per patient. The categories elementary motor, complex motor, somatosensory, and visual were reported most frequently, each in four patients. The after discharge annotation was used in three patients, the seizure annotation in four patients, the patient in doubt annotation in one patient, and the pay attention annotation was not used.

The usability testing was quantitatively analyzed using the SUS provided to five external end-users. The external end-users considered the usability of the new system good (mean SUS 78.0, SD 4.0). The five members of the co-design team also completed the questionnaire and considered the usability excellent (mean SUS 86.5, SD 10.8). The additional questions showed that half of the users could enter the categories in the iEEG software during stimulation (one user completely, four users over 50%) and the other five afterwards. During testing, clinicians experienced a short learning curve when annotating the categories of evoked clinical symptoms. All except one considered the analysis using the GUI more efficient than the workflow prior to implementation of the GUI. Eight users considered it less time-consuming, one user no difference, and one user more time-consuming compared to the work-up prior to implementation of the GUI. Eight users considered the new workflow easier, two as complex as before. Everybody indicated that the goal to standardize the workflow was achieved, nine users indicated that the goal to facilitate easy interpretation of the evoked clinical symptoms and seven users to save time analyzing the results was achieved. Eight users would not return to the old workflow of manual analysis of free text annotations.

### 3.3 The graphical user interface

**Fig. 3:**
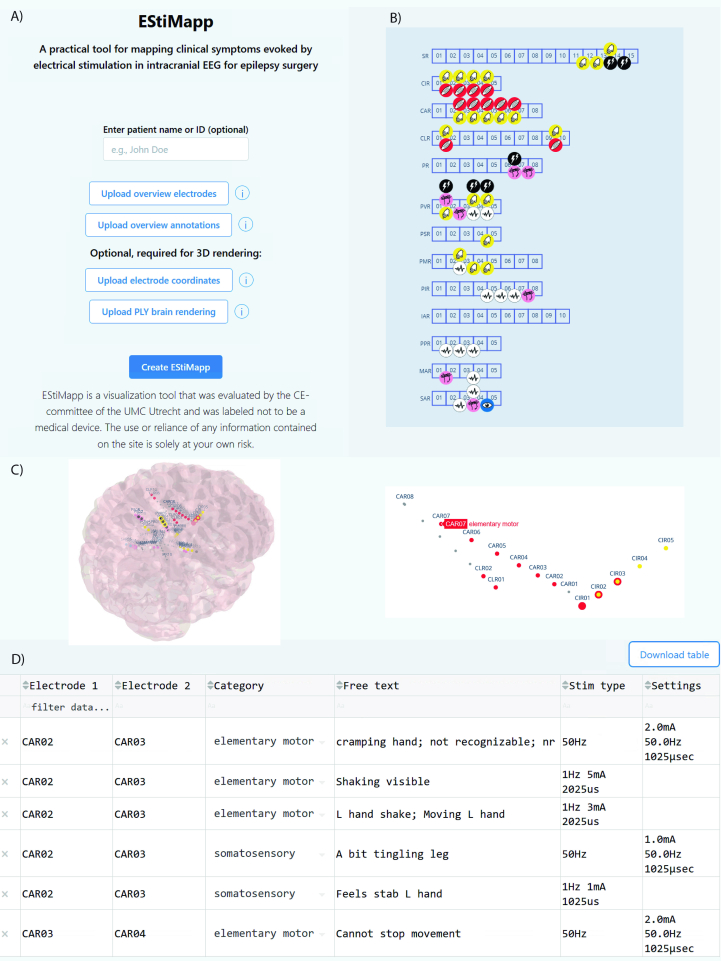
Impression of the GUI. A) The home page of the GUI, where the individual patient data or example data can be uploaded. B,C,D) The results page of the GUI. B) The 2D visualization where categories of evoked clinical symptoms are projected onto the patient specific electrode scheme. C) The 3D visualization where the corresponding color of the categories of evoked clinical symptoms are projected onto the electrode contacts in the brain. Left: Cortex and electrodes are visible. Right: Only electrodes are visible (opacity is set to zero). D) The table that shows the categories of evoked clinical symptoms, free text annotations and stimulation type per stimulation pair.

The final version of the GUI is shown in Fig. 3, and can be accessed via the link provided in section 2.4. Following the workflow explained in Fig. 2, the end-user uploads the patient specific electrode scheme, the annotations exported from the iEEG software, and optionally the entry and target coordinates of the electrodes and 3D PLY file of the cortex into the GUI. When the submit button is pressed, a results page is generated showing the *2D visualization* including the electrode scheme with projected icons representing categories of evoked clinical symptoms and a legend of the icons. Underneath, a table is shown with all annotated categories of clinical symptoms and free text annotations per stimulated electrode pair. When navigating to the table *3D visualization,* the 3D rendering is shown including the cortex and the implanted electrodes with the projected categories of clinical symptoms represented as colored markers. The 2D- and 3D visualization can be downloaded using the camera icon in the upper right part of the figure. The table can be downloaded using the download button in the upper right part of the table.

## 4 Discussion

We developed a clinical graphical user interface (GUI) to categorize and visualize evoked clinical symptoms during electrical stimulation on intracranial electrodes in 2D and 3D. The GUI was tested in clinical practice in the UMC Utrecht and end-users considered the GUI user-friendly. All indicated that the goal to standardize the workflow was achieved. This paper discusses the selection of clinical symptom categories, the co-design process, usability testing, and the presentation of the open-source GUI.

### 4.1 Categories of evoked clinical symptoms

The defined categories of evoked clinical symptoms were based on literature, including the recently updated position paper of the ILAE with guidelines for seizure classification (Beniczky et al., 2025). Compared to their descriptors for focal seizures, we made a few changes in order to adapt to the iEEG stimulation workflow. A difference between our categories and the ILAE classification is that we are using their subcategories of sensory phenomena. These sensory phenomena are commonly evoked during electrical stimulation and thus need to be expressed in detail. However, olfactory and gustatory are combined into one category because of the low prevalence (Asadi-Pooya, 2021; Ashalatha et al., 2018; Nakken et al., 2009; Russo et al., 2019). A similar argument as for the separation of sensory phenomena can be made for the cognitive and language phenomena; language mapping is an important aspect of electrical stimulation mapping and should be a separate category in our application.

The co-design team made a selection of categories of evoked symptoms and extra note types in order to balance essential information and simplicity. For example, laterality is not indicated but assumed to be as expected. In case of unexpected laterality, one could indicate that using the exclamation mark. In addition, for the focality of findings, the number of icons that appears in that region gives an impression of the spread of symptoms. Categorization modules for specific anatomical regions or symptoms could add more detail without the cost of overview.

### 4.2 Co-design process of the graphical user interface

The GUI is developed to answer a clinical demand to innovate the neurostimulation workflow for iEEG. The co-design team enabled us to closely collaborate with clinicians and to develop the GUI according to their needs with a focus on usability. We believe that this collaboration facilitates implementation of GUIs in the field and can bridge the gap between research and clinical practice. Especially for medical diagnostics with center specific workflows like electrical stimulation mapping, co-designing can play an important role in developing this niche expertise field. Job disciplines such as technical medicine can initiate, guide and accelerate these technical developments and novel innovation strategies in healthcare (The NVvTG – NVvTG.nl, 2026).

### 4.3 Evaluation of the graphical user interface

The System Usability Scale showed that the co-design team considered the usability to be ‘excellent’ and the external end-users to be ‘good’. The workflow using the GUI is considered more efficient, less time-consuming, and makes the work easier compared to prior to implementation of the GUI. The predefined goals to standardize the workflow, facilitate easy interpretation of the evoked clinical symptoms, and save time analyzing the results are achieved. The results show that the new workflow using the GUI is well-accepted in clinical practice. Usability testing was partly done by members of the co-design team, which may limit the generalizability. The usability score of the five end-users not in the co-design team shows that this effect is limited and that the GUI is suitable for the workflow in both participating centers. More feedback should be gathered to test compliance with other iEEG software.

### 4.4 Comparison to state of the art

Although four other GUIs, IntrAnat, YAEL, RAVE, and gTEC cortiQPRO are known in the field, each lacks standardized features or functionalities that our GUI provides. To elaborate, IntrAnat is developed for research purposes and covers not only visualization, but also localization of electrodes and group analysis using projection on brain atlases (Deman et al., 2018). Clinicians using this GUI can add categories of clinical symptoms, but since they define the labels themselves, these labels lack standardization. In addition, the labelling of electrodes has to be carried out manually. YAEL showed similar functionalities as IntrAnat and is currently replaced by RAVE (Wang et al., 2023). RAVE focuses on data analysis and visualizes the analyzed characteristics on a cortical surface (Magnotti et al., 2020). Moreover, this GUI also lacks a guideline to categorize evoked clinical symptoms. These three GUIs solely provide 3D visualization and co-registered MRI slices. In contrast, our GUI's 2D electrode projection can assist in creating a quick overview of the evoked clinical symptoms, setting it apart from the other three GUIs mentioned. Finally, gTEC cortiQPRO offers an electrical stimulation mapping tool, but requires the purchase of their software and hardware system and thus could not be tested elaborately. An overview of the specifications of all GUIs can be found in Appendix C.

### 4.5 Strengths and limitations

Our GUI offers several strengths that facilitate standardization of the intracranial stimulation workflow and enable straightforward interpretation of evoked clinical symptoms, which can aid in decision making for determining the resection area during presurgical evaluation. Although the neurostimulation workflow varies between hospitals, we offer an open-source and web-based tool that requires minimal input data to allow for widespread clinical implementation. In addition to accessibility, for local installation of the GUI the patient data is processed locally to prevent any privacy risks. Hence, the GUI is considered easy to implement and safe. Furthermore, because of the reduction of manual tasks, the proposed method is more time saving, less error-prone and easier to use compared to the current clinical practice. Apart from a 2D overview, 3D visualization is implemented, which is helpful to readily match clinical symptoms with the anatomical location of stimulated electrode pairs.

During testing, clinicians experienced a short learning curve when annotating the categories of evoked clinical symptoms. In the beginning, annotating the categories afterwards may be more feasible than during stimulation. To assist in the classification of clinical symptoms, the table with annotations could be made interactive so the categories can also be added within the GUI. The quality of the visualization is dependent on the input of the clinician. The GUI is solely a visualization tool and should be used as such. Another challenge regarding the categories of evoked clinical symptoms is that the categories are less detailed than free text annotations. On the other hand, the categories can help to give a clear overview and enable us to compare results between patients. The detailed information (free text annotations) is depicted in the table below the visualization. Furthermore, a notable observation is that the 3D visualization indicates the approximate location of the electrodes in the brain, but deviations from the exact location may occur. In general, although we tested the functionality of the GUI thoroughly, we would like to raise awareness to the risk of erroneous presentation of results in the software that could possibly lead to medical consequences. As for any medical innovation, the benefits of using the software and visualizing medical data should be weighted to the risk of using open source software. This risk occurs if people would use the tool for medical advice rather than facilitating visualization as it is intended for. Another risk occurs with implementing automated classification, e.g., using a large language model to redirect clinician's notes. This last step is as attractive as potentially harmful. A broader discussion how to safely implement these tools could raise awareness and be a first step to implementation guidelines.

### 4.6 Future perspectives

The GUI is implemented in clinical practice in UMC Utrecht and can be adopted by any epilepsy center performing electrical stimulation on intracranial electrodes. A small development could enable usage for seizure classification in video EEG monitoring. In this way, the EEG can be interpreted in a standardized way and the results can be used for those who are followed up with iEEG. Implementation of the GUI in other centers helps to align iEEG neurostimulation research, for example by enabling to create a functional brain atlas. Balestrini et al., Sarubbo et al. and Michalak et al. have developed functional atlases (Balestrini et al., 2015; Michalak et al., 2025; Sarubbo et al., 2020), but the center-specific workflows have impeded the next step towards a multicenter approach (Ritaccio et al., 2018). The proposed workflow with the GUI could be the starting point for this enhanced functional atlas. Subsequently, the atlas could highlight deviating findings in a patient or predict eloquent areas when neurostimulation does not evoke any symptoms. In clinical practice, projecting a resection proposal to the normative map gives insight in possibly involved eloquent areas.

The GUI could be further developed by adding detailed categorization modules for stimulation in specific anatomical areas, such as the insula, the visual cortex, the sensorimotor cortex, Broca's and Wernicke's area. Similar modules for negative motor, language and cognition symptoms would enable to study these phenomena in more detail. In addition, free text annotations could be interpreted automatically into the categories of clinical symptoms by a large language model. Notable is that this automatic interpretation would turn the GUI into a medical device and that interpretation of the clinical symptoms shifts from clinician to algorithm. While the current version focuses on intuitive visualization within the ESM context, future development could enable interoperability with established imaging pipelines to facilitate multimodal interpretation. Besides visualizing the clinical interpretation of evoked clinical symptoms, the EEG during electrical stimulation could be analyzed; for example high gamma mapping is considered to play an important role in task-related brain activity (Darvas et al., 2009). In the future, the workflow enables us to focus our time and effort on the patients, instead of on administrative tasks. By relating anatomical location to symptomatology, we can further develop our understanding of eloquent and epileptic brain areas.

## Declaration of competing interest

The authors declare that they have no known competing financial interests or personal relationships that could have appeared to influence the work reported in this paper.

## Figures and Tables

**TABLE A1:** CO-DESIGN ACTIVITIES.

Activity	Co-design phase	Objectives
Brainstorm 1 *26 September 2024*	1) Define the problem of the end-users 2) Understand the context of use and lived experience	Introduction to the project and co-design process; Identify advantages and disadvantages of the current clinical practice and proposed workflow.
Brainstorm 2 *19 December 2024*	2) Understand the context of use and lived experience 3) Build a design consensus	Discuss required outcomes of neurostimulation; Inventorize desired specifications; Define categories of evoked clinical symptoms. See Table 1, Table 2.
Brainstorm 3 *6 February 2025*	3) Build a design consensus 4) Establish design specifications and do pilot-testing	Define annotations of categories; Discuss visual representation of categories; See Fig. 2.
Testing prototype *23 June to 19 August 2025*	4) Establish design specifications and do pilot-testing	Test the functionalities of the GUI (2D plot and table) for three patients; Usability testing of the prototype via user interviews.
Brainstorm 4 *21 August 2025*	4) Establish design specifications and do pilot-testing	Review draft version of GUI.
Testing GUI *30 September to 29 October 2025*	4) Establish design specifications and do pilot-testing	Test the functionalities of the GUI (2D and 3D plot and table) for seven patients; Usability testing of the prototype via user interviews and questionnaires (Appendix B); See Fig. 3.

**Table:** 

	Strongly disagree	Disagree	Neither agree nor disagree	Agree	Strongly agree
I think that I would like to use the GUI frequently	O	O	O	O	O
I thought the GUI was easy to use	O	O	O	O	O
I felt very confident using the GUI	O	O	O	O	O
I found the various functions of the GUI were well integrated	O	O	O	O	O
I would imagine that most people would learn to use the GUI very quickly	O	O	O	O	O
I found the GUI unnecessarily complex	O	O	O	O	O
I found the GUI very cumbersome to use	O	O	O	O	O
I thought there was too much inconsistency in the GUI	O	O	O	O	O
I needed to learn a lot of things before I could get going using the GUI	O	O	O	O	O
I think that I would need the support of a technical person to be able to use the GUI	O	O	O	O	O

**Table B1:** Comparison of GUI platforms. iEEG = intracranial electroencephalogram; ESM = electrical stimulation mapping; FDA = Food and Drug Administration.

Feature Category	IntrAnat	YAEL	RAVE	gTEC cortiQPRO	EStiMapp
Official Website / Download Link	GitHub: https://github.com/IntrAnatSEEGSoftware/IntrAnat	https://yael.wiki	https://rave.wiki	https://www.gtec.at/product/cortiq-pro/	https://estimapp.onrender.com
Primary Purpose	Electrode localization, multimodal visualization, group analysis	Electrode localization and visualization	iEEG analysis + visualization	Cortical mapping system	Standardized ESM + visualization
Open-Source Status	Free and open-source	Free and open-source	Free and open-source	Commercial	Free and open-source
Supported File Formats	TRC (Micromed), multimodal MRI/CT/PET inputs	Not specified	Matlab, EDF, BIDS-iEEG, HDF5	Not specified	Excel, MicroMed CSV export, PLY
Clinical vs Research Use	Research-only	Research + clinical visualization	Research + clinical visualization	Clinical medical device (FDA-listed)	Research + clinical visualization
Symptom Categorization	Manual, non-standardized	Manual, non-standardized	No guideline	Not specified	Standardized categories
Visualization Features	3D + MRI/CT/PET	3D	3D cortical surface	3D, real-time high-gamma mapping	2D projection +3D
Regulatory Status	Not specified	Not specified	Not specified	FDA device listing (cortiQ PRO 8060)	Not registered as medical device

## Appendix B Questionnaire usability testing.

1. How many years have you been in practice (following residency and/or fellowship)?

o Less than 5 years

o 6–10 years

o 11-15 years

o 16–20 years

o Greater than 20 years

2. What was your clinical FTE during the period Jan 2020 - Jan 2025? Choose the closest option below.

o 0.25

o 0.50

o 0.75

o 1.0

3. System Usability Scale

4. With regard to entering the categories of evoked clinical symptoms, was it feasible to fill in the categories during stimulation?

o Yes, I could completely enter in the categories during stimulation

o Yes, I could enter most of the categories during stimulation

o No, I did not have enough time to define the category during stimulation, so I entered the categories afterwards

o No, I could not define the right category

5. With regard to analyzing the evoked clinical symptoms afterwards, how efficient was the analysis using the GUI compared to prior to implementation of the GUI?

o Analysis using the GUI is less efficient
o Analysis using the GUI is more efficient
o No difference in efficiency

6. With regard to analyzing the evoked clinical symptoms afterwards, how time-consuming was the analysis using the GUI compared to prior to implementation of the GUI?

o Analysis using the GUI takes less time
o Analysis using the GUI takes more time

o No difference in time

7. The new workflow (annotating categories and using the GUI) makes the work.

o … easier

o … as complex as before

o … more difficult

8. The GUI was designed as a group effort with three goals: 1) standardize the workflow; 2) easy interpretation of the evoked clinical symptoms; 3) save time analyzing the results. Please indicate which of the goals you think are achieved.

□ standardize the workflow

□ easy interpretation of the evoked clinical symptoms

□ save time analyzing the results

□ None of the goals are achieved

9. Given the option of returning to the manual analysis of free text annotations, would you return to that option?

o Yes

o No

## Appendix C Supplementary data

Supplementary material EStiMapp user guideline
