# Supplementary material for: EStiMapp: A practical tool for mapping clinical symptoms evoked by electrical stimulation in intracranial EEG for epilepsy surgery
Source: Clin Neurophysiol Pract. 2026 Jun 12;11:492–502. doi: 10.1016/j.cnp.2026.06.006 (PMC13333301; doi:10.1016/j.cnp.2026.06.006)
Supplement: Supplementary material — EStiMapp user guideline [file mmc1.docx]

# S1 - EStiMapp user guideline

This is a step-by-step guide to use EStiMapp, a graphical user interface to visualize clinical symptoms evoked by electrical stimulation in intracranial EEG for epilepsy surgery. Step 1 describes how to annotate the iEEG, step 2 describes how to use EStiMapp.

Are using the example patient data? You can skip step 1 and 2 and go to step 3.

The source code is publicly available on GitHub: <https://github.com/UMCU-EpiLAB/umcuEpi_estimapp.git>

The application is available on: <https://estimapp.onrender.com>

The data of one example patient with stereo EEG is available on DataverseNL: <https://doi.org/10.34894/KMT3VI>

## Annotate evoked clinical symptoms

If you are using MicroMed, you can enter the annotations directly into the EEG and export the annotations afterwards, see step 1a. Probably, for other EEG systems a similar pipeline can be followed. If not, follow step 1b to annotate via Excel.

### MicroMed users

For every file, you need to indicate the start and end of a stimulation period.

Annotation before start of stimulation period:

- *Stim_on;[stimulation type]*
- For example: *Stim_on;1Hz*

Annotation after end of stimulation period:

- *Stim_off;*

Please note that you need to enter these annotations per stimulation type if you have multiple in one file.

For every stimulated electrode pair, the EEG system automatically adds an annotation (e.g. *AR1 – AR2*). If clinical symptoms are evoked, enter the abbreviation of the corresponding category of clinical symptoms as annotation to the EEG. The abbreviations and descriptions of the categories can be found in Table 1. Make sure to enter the abbreviation of the category after the stimulated electrode pair annotation and before the next stimulus. You can enter multiple categories by making multiple annotations.

When you are finished, export the annotations in MicroMed via *File > Export > Export Note\Events.* Choose a folder on your pc, give the file a name and add ‘.csv’, e.g. *annotations.csv*, and click save. A pop-up appears, select all export options and export by time, see the screenshot below. Click on *Begin*.


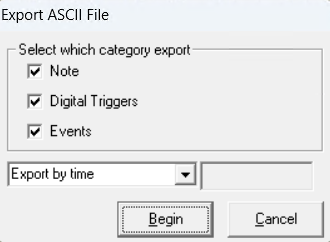


### Annotate via Excel

Create a new excel file. On the first line, type: *Comment*

EStiMapp reads the excel from top to bottom in chronological order.

For every stimulation type, you need to indicate the start and end of a stimulation period.

Annotation before start of stimulation period:

- *Stim_on;[stimulation type]*
- For example: *Stim_on;1Hz*

Annotation after end of stimulation period:

- *Stim_off;*

Every (symptom evoking) stimulated pair of electrodes should be placed in a cell, followed by the notes corresponding to this electrode pair. Notes can be free text annotations describing clinical symptoms, or the abbreviation of a corresponding category. The abbreviations and descriptions of the categories can be found in Table 1. Make sure to enter the abbreviation of the category below the stimulated electrode pair annotation and above the next stimulus. You can enter multiple categories by making multiple annotations. See example Figure 1 line 9-13, where ‘*dizzy’*, ‘*sz’*, ‘*vest’*, and ‘*recognizable*’ belong to PR06 – PR07 above.

Optional: if you want to include the stimulation parameters, add them to the stimulation type in *Stim_on;* or to the electrode pairs:

- *PR06-PR07 3.0mA 50.0Hz 1025µsec*

Save the excel file.


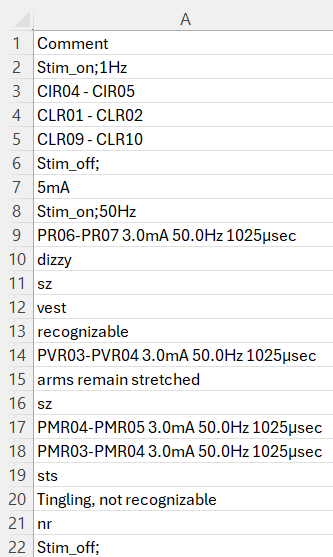


**Figure 1:** Example of an excel file containing annotations.

**Table 1:** Clinical symptoms categories and corresponding abbreviations and icons.

| Category | Abbreviation | Description of category |
| --- | --- | --- |
| 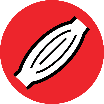Elementary motor | em | - Myoclonic: sudden, irregulare muscle jerks of short duration (<400 ms), prominently affecting the shoulders and proximal arms, but can be focal; - Clonic: repetitive, short contractions of agonist muscle groups, recurring at regular intervals of 0.2-5 per second, usually affecting the distal extremity or face; - Tonic: sustained contraction of one or more muscle groups lasting at least 3 seconds and leading to posturing of the limbs and/or trunk, usually affecting proximal muscles in a bilateral fashion; - Versive: sustained, forced, unnatural turning of the head or eyes to one side, having a tonic or clonic quality. Typically, the angle of the mouth is deviated to the same side and the head is hyperextended; - Tonic-clonic movements: sequence of a generalized tonic contraction followed by clonic activity lasting 1-2 minutes. |
| 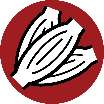  Complex motor | cm | - Hypermotor: repetitive complex movements involving the proximal limbs and trunk that are rapid and violent in nature. Motor activity simulates normal movements inappropriate for the situation (e.g. thrashing, rocking, jumping, waving, bicycling, kicking). Vocalisation, laughter and crying are commonly observed; - Automotor: repetitive, stereotyped, semi-purposeful motor behaviours, involving primarily the distal limbs, mouth and tongue including mastication, swallowing, lip smacking, blowing, whistling, and kissing. Those involving the distal extremities include fumbling, picking, and gesticulating movements; - Gelastic: brief periods of laughter or grimacing with or without the subjective feeling of mirth. |
| 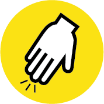Somato-sensory | sts | - Anesthesia, e.g. numbness - Paresthesia, an abnormal sensation, but not unpleasant. e.g. tingling, sensation of swelling or shrinking, sensation of movement of body parts - Dysesthesia, an unpleasant, abnormal sense of touch. E.g. painful sensation including burning, itch, pricking, muscle tearing, pain in a limb, electrical shock-like feelings - Whole-body sensation, supernumary phantom limb, missing limb, reduced localizing and lateralizing value of sensations affecting the entire body - Thermic dispersion/sensation, shivering, goosebumps |
| 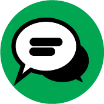  Language | la | - Cognitive, including aphasia and paraphasic errors - Motor, including dysarthria and speech motor arrest - Memory, including amnestic anomia |
| 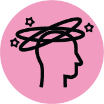Vestibular | vest | Illusions of rotation or translation in all planes, or indefinable feelings of body motion, spinning sensation, dizzy feeling. |
| 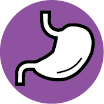Autonomic | auto | - Cardiorespiratory, e.g. palpitations, tachycardia, chest tightness and shortness of breath, choking, unique sensation of the heart; - Gastrointestinal, e.g. sialorrhea, vomiting, retching, nausea, rising, abdominal pressure, butterflies in the stomach, emptiness, tightness, malaise, pain, hunger, swelling, indescribable discomfort in the abdominal or periumbilical area that can be static or rise to chest and throat - Genitourinary, e.g. genital sensations (often painful, unpleasant sensations associated with fear), urinary urge - Cutaneous, e.g. generalized feeling of warmth or cold, goosebumps - Sexual, erotic thoughts and feelings and pleasurable genital sensations, sexual arousal, erection, and orgasm that are occasionally accompanied by viscerosensory phenomenon (i.e., vulvovaginal secretion) - Lacrimation, flushing |
| 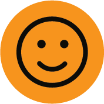  Affective | aff | - Positive feelings, e.g. happiness, pleasure, delight, excitement, joy, elation, euphoria, satisfaction - Negative feelings, e.g. dysphoria, sadness, depression, anger, fear, anxiety, nervousness, impending doom, elation |
| 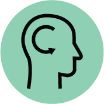  Cognitive | cog | - Distortions of familiarity, e.g. déjà vu, jamais vu - Illusions (incorrect and distorted perception of something objective), hallucinations (perceptual experience that occurs in the absence of a corresponding objective stimulus), multisensorial hallucinations including revocation of complex memories, out-of-body experiences - Forced thought, subjective experience of being confused (mixed-up thoughts) |
| 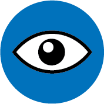  Visual | vis | - Simple visual auras, e.g. static, flashing, or moving lights in different shapes and colors, elementary hallucinations, contralateral eye deviation, blinking, a sensation of eye movement, nystagmoid eye movements, phosphenes (luminous floating stars, zigzags, swirls, spirals, squiggles, and other shapes) - Complex visual auras, e.g. people, scenes, objects, and optical illusions - Blurry vision or visual motions, transitory blindness - Visual distortions, e.g. micropsia, macropsia, metamorphopsia, and palinopsia |
| 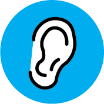  Auditory | audi | - Simple auditory hallucinations, e.g. ringing and buzzing sounds, tinnitus, clicking, whistling, humming - Complex auditory hallucinations, e.g. voices and music, meaningful sounds - Positive illusions, e.g. increased loudness and altered frequency or tempo, pitch, distortion, echo - Negative illusions, e.g. auditory agnosia and hypoacusia (reduction of external sounds until a temporary deafness) |
| 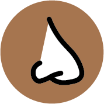  Olfactory or gustatory | og | - Olfactory are typically (unpleasant) smelling sensations - Gustatory are tasting sensations |
| 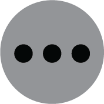  Other | ot | Clinical symptom does not fit any category |
| 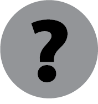  Patient in doubt | ? | Patient is in doubt about experience |
| 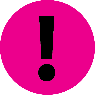  Pay attention | ! | Unexpected finding, pay attention |
| 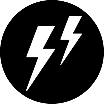  Seizure | sz | Stimulated seizure |
| 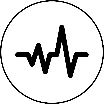  After discharge | ad | Stimulated after discharge |

## Prepare file upload

Besides the annotations, an electrode overview is required to use the app. For the optional 3D visualization, a 3D rendering of the brain and the electrode coordinates are required.

### Electrode overview

Create an excel file that contains all electrode contacts, similar to the file of the example patient:


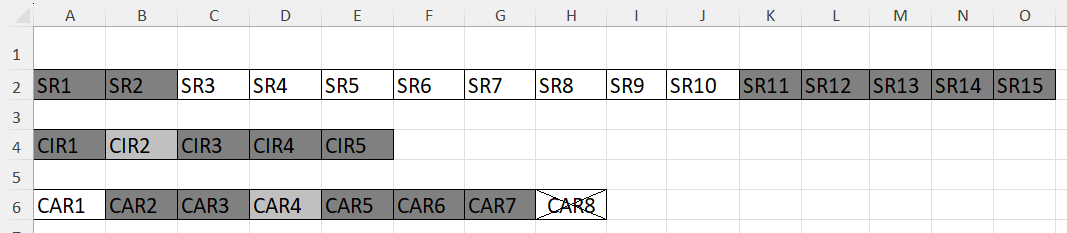


You can ignore the different gray highlight colors.

### Electrode coordinates (for 3D visualization)

The electrodes are projected onto the cortex rendering. In order to do so, the electrode name, the number of contacts, entry- and target xyz-coordinates are required. Create an excel file that contains all information, similar to the file of the example patient:


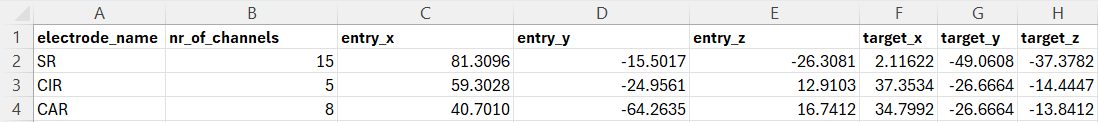


###
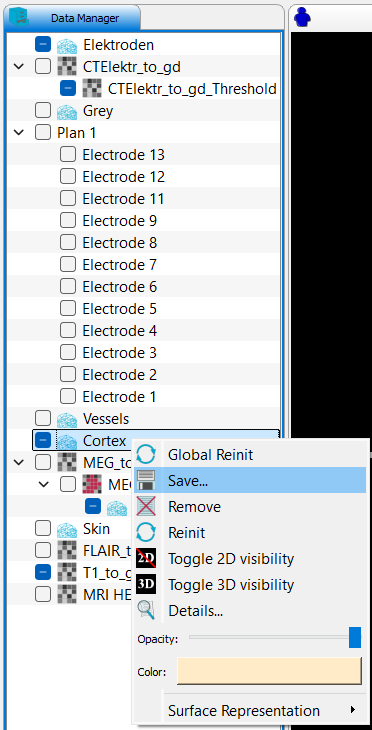
Cortex rendering (for 3D visualization)

A PLY-file of the cortex is required to make the 3D visualization. This can for example be extracted from EpiNav.

- Open a patient file
- Select the cortex rendering in the Data Manager, right click on Save (see screenshot)
- Choose a folder on your pc, give the file a name and select save as .PLY

For more information about EpiNav, please find:

*Vakharia, V.N., Sparks, R., Miserocchi, A. et al. Computer-Assisted Planning for Stereoelectroencephalography (SEEG). Neurotherapeutics****16****, 1183–1197 (2019). https://doi.org/10.1007/s13311-019-00774-9*

## Create EStiMapp

Home page

1. Go to your local hostpage or to the website <https://estimapp.onrender.com>
2. Optional: enter patient name or ID
3. Upload overview electrodes (step 2a)
   1. Example patient file name: *Electrodes overview.xlsx*
4. Upload overview annotations (step 1), one or multiple files.
   1. Example patient file name: *Annotations_*Hz_file*.csv*
5. Optional: upload electrode coordinates for 3D visualization (step 2b)
   1. Example patient file name: *Electrode_coordinates_3D.xlsx*
6. Optional: upload cortex rendering for 3D visualization (step 2c)
   1. Example patient file name: *Cortex_3D.ply*
7. Press ‘*Create EStiMapp’*

Results page

- Navigate between 2D and 3D visualizations using the buttons. A table with information about evoked clinical symptoms is shown below either figure.
- 2D: Categories of evoked symptoms are projected onto the electrode overview. The icons are explained in the legend.
- 3D: Categories of evoked symptoms are projected onto the electrodes. The colors of the electrodes are linked to the colors of the icons in the legend. If you hover over the electrode, the category label appears.
- Table: contains the stimulated electrodes, category, free text annotations, stimulation type and settings. You can remove lines, and change the category or free text annotations and download the (altered) table. Changes in the table do not affect the visualizations.
